# Retraction: colon and rectal surgery for cancer without mechanical bowel preparation: one-center randomized prospective trial

**DOI:** 10.1186/1477-7819-10-196

**Published:** 2012-09-20

**Authors:** Stefano Scabini, Edoardo Rimini, Emanuele Romairone, Renato Scordamaglia, Giampiero Damiani, Davide Pertile, Valter Ferrando

**Affiliations:** 1 Unit of Surgical Oncology, Department of Emato-Oncology, San Martino Hospital, Genoa, Italy

## 

Retraction

The authors have retracted this article [1] because it contains large portions of text that have been duplicated from another article previously published in * Annals of Surgery *[2]. The authors apologise to the Editors and readers as well as the authors of the original article.
